# Utilising community volunteers can increase the detection and referral of Buruli ulcer cases in endemic communities in Southeast, Nigeria

**DOI:** 10.1186/s40794-022-00181-7

**Published:** 2022-11-01

**Authors:** Chihurumnanya Alo, Ijeoma Nkem Okedo-Alex, Ifeyinwa Chizoba Akamike, Adaoha Pearl Agu, Ifeyinwa Maureen Okeke, Chidinma Ihuoma Amuzie, Nneamaka C. Alo

**Affiliations:** 1 Department of Community Medicine, Faculty of Medicine, Ebonyi State University, Ebonyi State, Abakaliki, Nigeria; 2 Department of Community Medicine, Alex Ekwueme Federal University Teaching Hospital Abakaliki, Abakaliki, Ebonyi State Nigeria; 3 African Institute for Health Policy and Health Systems, Ebonyi State University (EBSU) Abakaliki, Abakaliki,, Nigeria; 4 Department of Community Medicine, Ebonyi State University Abakaliki, Abakaliki, Ebonyi State Nigeria; 5 Department of Community Medicine, Federal Medical Centre, Umuahia, Abia State Nigeria; 6 Nigeria Field Epidemiology Training Program, Abuja, Nigeria; 7 Department of Family Medicine, Alex Ekwueme Federal University Teaching Hospital Abakaliki, Abakalik, Ebonyi State Nigeria

**Keywords:** Buruli ulcer, Community volunteers, Case detection, Hospital referral, Neglected tropical disease, Nigeria

## Abstract

**Background:**

Buruli ulcer (BU) is a debilitating neglected tropical disease which causes disability and mostly affects inhabitants in impoverished settings where access to medical care is challenging. This study aims to determine the effect of training community members as volunteers for or in the detection and referral of people who have Buruli ulcer to the hospital.

**Methods:**

The following study is a before and after study in the BU-endemic Local Government Areas (LGA) of Ebonyi State. A cluster random sampling technique was used to select 90 volunteers from three LGAs (30 from each LGA). In each LGA, the volunteers underwent a one-day training and six months field work to identify all those who have any form of ulcer on any part of their bodies. A short questionnaire was used to capture socio-demographic characteristics of the patient, site of the ulcer, duration of the ulcer, initial appearance of the ulcer, referral to hospital, result of laboratory investigation, and treatment received. The data was analysed using the Statistical Package for Social Sciences (SPSS) for Microsoft Windows version 20 software. The Z test statistic was used to compare the number of referred BU patients before and after the intervention by LGA. The Chi square test was used to examine the association between the dependent and independent variables.

**Results:**

The mean age of volunteers was 39 ± 9.5 while mean age of the patients was 42.3 ± 17.1. Most of the ulcers were on the legs (79.4%) and lasted 1–5 years (65.6%). There was a significant increase in the proportion of BU suspects identified by the community volunteers in all 3 LGAs (Afikpo north (*p* =  < 0.001), Abakaliki (*p* = 0.02), Ikwo (*p* = 0.001). The duration of the ulcer was associated with the detection and referral of the patients with higher levels of detection and referral among those whose ulcer had lasted 1–5 years in two of the LGAs (*P* < 0.001).

**Conclusion:**

We recommend that program managers and stakeholders integrate and scale up the services of trained community health volunteers for the rapid detection of Buruli ulcer cases in rural endemic communities. Awareness and sensitization campaigns on BU preventive measures should be intensified.

## Introduction

Buruli ulcer (BU), caused by *Mycobacterium ulcerans*, is an emerging neglected tropical disease [1]. *M. ulcerans* is part of a large group of environmental mycobacteria which is endemic in many countries, especially countries around the tropical rain forest [1]. Transmission of BU infections occur through a bruise on the skin or mild traumatic injuries after coming into contact with contaminated water, soil, or vegetation [1]. Buruli Ulcer mostly affects children and it progresses from painless nodules to large, undermined ulcerative lesions that heal spontaneously though slowly [1]. The disease is accompanied by few systemic symptoms however secondary infections can result in sepsis or tetanus leading to severe systemic disease or death [1]. Extensive scarring can lead to contractures of the limbs, blindness, and other adverse complications. It is the third most common mycobacterial disease after tuberculosis and leprosy in immuno-competent persons [1]. The disease has been reported in more than 30 countries worldwide, but the highest patient load is in West Africa. Within the endemic countries, BU occurs in foci typically affecting inhabitants of impoverished and rural settings where access to medical care presents a great challenge. The lesions are divided into three categories: category I: a single lesion < 5 cm in diameter; category II: a single lesion 5-15 cm in diameter; and category III: single lesion > 15 cm in diameter including multiple lesions, lesions at critical sites such as eye, breasts, genitalia and osteomyelitis [2]. If not treated early, the nodule gradually enlarges and erodes through the skin surface, leaving a well-demarcated ulcer with a necrotic slough in the base and widely undermined edges, which are the hallmarks of the disease [3]. Prolonged delay might lead to bone involvement and functional disabilities such as amputation of limbs and vital organs such as the eye [3]. Despite the belief that *M*. *ulcerans* can be acquired from environmental sources in endemic areas, the exact mode of transmission is currently incompletely understood. Risk factors identified that increased susceptibility for BU involves being aged below 15 years or over 49 years, poor hygiene of existing wounds, living close to stagnant water, insect bites, the water sources used, and the activities near them [4, 5].

The prevalence of BU in Nigeria is uncertain as there has not been any documented National prevalence of the disease. In regions of the country or individual states, cases that have been reported are those that present to the hospitals. The National worker’s manual only states that it has been confirmed that Nigeria is an endemic country with many states already involved [4]. The inability to quantify the magnitude of this debilitating disease contributes to BU being continuously listed with the neglected tropical diseases. This will further complicate the allocation of adequate health resources while working towards its control and elimination. Many cases that are reported at the formal health sector for treatment present late and various reasons have been attributed to this [5]. It has been observed that the case fatality among those affected by BU is low however they experience a high degree of morbidity or disability [6].

It has been opined that the primary strategy for control is early detection using community volunteers. Researchers in Benin, collected data to understand the role of the different referral systems on the stage of disease at presentation in the hospital and the diagnostic precision [7]. About a quarter of the patients were referred to the hospital by the community health volunteers and they concluded that community health volunteers referred patients more frequently in an earlier stage of disease [7]. In the absence of a full understanding of the mode of transmission or of a vaccine, the main objective of BU control is to reduce the morbidity and disability associated with the disease. Drug treatment for BU consists of a combination of antibiotics given for eight weeks. The current World Health Organization (WHO)-recommended regimen is rifampicin (10 mg/kg once daily, oral tablet) combined with streptomycin (15 mg/kg once daily, intramuscular injection) [8]. Wound care is performed three times a week to daily depending on the severity of the wound [2]. It has been opined that Information, Education, and Communication (IEC) intervention will encourage early case detection and treatment with the assumption that once people gain knowledge they will take the appropriate action to assess treatment early. In this direction Ackumey et al. [9] stated that intensifying health education and surveillance will create awareness and encourage early treatment. The aim of this study is to determine the effect of training community members as volunteers on the detection and referral of people who have Buruli ulcer to the hospital.

## Methods

### Study location

This study was carried out in Ebonyi State, South Eastern Nigeria. The state got its name from the Ebonyi River whose tributaries run across different communities in the state. The people of the state are agrarian in nature probably occasioned by the swampy nature of the land and presence of bodies of water. The state is made up 13 local government areas (LGAs) and has Abakaliki as its state capital. The National Tuberculosis, Leprosy and Buruli Ulcer Control Program (NTBLCP) is located in Abakaliki under the state ministry of health. They carry out BU control activities such as identification of cases, diagnosis and treatment of cases and referral when necessary. The NTBLCP has a unit in the state ministry of health that oversees all the control activity of BU in the state. The unit identifies BU through the BU LGA focal person who occasionally visits the community. Because of the ulcer, patients may also visit the health facility in the community by themselves and get enrolled into treatment through the routine disease notification system.

### Study population

The study population comprises both community members who have ulcer(s) anywhere on their body and community members who are willing to volunteer, be trained to identify the cases of ulcers, and refer them to the hospital. The inclusion criteria for the community volunteers is as follows: any community member who has lived for more than 5 years in the community and who may either be a traditional bone setter, traditional birth attendant, patent medicine dealer, retired teacher, or a retired health worker.

### Study design

This is a before and after study where the communities serve as their own controls.

### Sampling

The sampling method used is a cluster random sampling technique. A Local Government Area (LGA) formed a cluster and three LGAs were selected using balloting technique. Thirty Community volunteers were recruited in each of the LGA and they were trained to identify all those who have any form of ulcer on any part of the body.

### Data collection

The data collection tool was a checklist designed to capture sociodemographic characteristics of the patient, site of the ulcer, duration of the ulcer, initial appearance of the ulcer, referral to hospital, result of laboratory investigation, and treatment received.

At baseline, the number of confirmed BU cases identified by the last quarterly report of the NTBLCP in the selected LGAs was assessed. Information on the number of BU cases that were identified, bacteriologically confirmed, and placed on treatment through the routine disease notification system. Thereafter, members of the community who volunteered to be part of the study received a one-day training per LGA on how to identify cases of BU, enroll them into the study, and refer them to hospital through the BU control program focal person for the LGA. The training was carried out in the three LGAs at different dates. The National Tuberculosis, Leprosy & Buruli ulcer Management & Control Guidelines 2015 were used to adapt and bring out the salient issues that would help the volunteers understand their role in the community during the months following. Handbills, with pictures of Buruli ulcer lesion, were used to enable the volunteers to understand the different presentations of Buruli Ulcer. The volunteers practiced how to fill the short questionnaire and became acquainted with how to work with it in the field.

During the intervention phase, which lasted for six months, community volunteers and the researchers met once a month in a designated health facility in the LGA to review their activities and to retrieve the data they collected from the field. During this period, the community volunteers also visited churches, schools, and village meetings to create awareness about BU and the benefits of going to the hospital to get the help of orthodox medicine. From these activities, each patient they identified or who went to the volunteer on his or her own accord completed the short questionnaire. The patients were then invited to the meeting of the researchers and community volunteers. On the scheduled day of the meeting, the volunteers submitted the completed questionnaires and one of the researchers transferred the data to an excel sheet. The identified patients travelled with the volunteers to the health facility and were enrolled into the treatment register of the State BU control unit. After the enrollment, the Laboratory focal person for the state BU control team collected a swab sample from the ulcer. The sample was used for microbiological diagnosis according to the National guideline in the diagnosis of BU. At this point, the patient was followed up according to the treatment guidelines in the National control program handbook for the control of BU. At the end of the sixth months, the number of patients identified using community volunteers was compared to that reported in the register of the state NTBLCP.

### Statistical analysis

The dependent variable was the proportion of BU cases referred while the socio-demographic and BU characteristics were the independent variables. The data was analyzed using the Statistical Package for Social Sciences (SPSS) for Microsoft Windows version 20 software. Frequencies and proportions for categorical variables were calculated while mean and standard deviations were computed for numerical variables. The Z test statistic was used to compare the number of refereed BU patients before and after the intervention by LGA. The Chi square test was used to examine the association between the dependent and independent variables.

## Results

There were 30 volunteers in each LGA giving a total of 90 out of which males were 47 and females were 43. Their mean age was 39 ± 9.5 (Table 1).

**Table 1:** Socio-demographic characteristics of the community volunteers (*n* = 90)

Variable	Frequency (%)
**Community volunteers in each LGA**	
Afikpo	30 (100.0)
Abakaliki	30 (100.0)
Ikwo	30
**Sex**	
Male	47(47%)
Female	43(43%)
**Mean age**	39 ± 9.5

Table 2 shows the socio-demographic characteristics of the patients identified to have Buruli ulcer in all three LGAs. The mean age of the patients was 42.3 ± 17.1. Adults constituted 80.9% of the patients and 50.4% of them were males. A third of the patients had primary (33.6%) and secondary education (32.1%). Slightly less than half (43.5%) of the patients were farmers.

**Table 2:** Socio-demographic characteristics of the patients identified to have Buruli ulcer in all three LGAs

Variable	Frequency *n* = 131	Percentage
Mean Age(standard deviation)	42.3 ± 17.1	
**Age groups**		
Children	15	11.5
Adults	106	80.9
Older adults	10	7.6
**Sex**		
Female	65	49.6
Male	66	50.4
**Marital status**		
Married	89	67.9
Single	30	22.9
Widowed	12	9.2
**Educational status**		
None	33	25.2
Primary	44	33.6
Secondary	42	32.1
Tertiary	12	9.2
**Occupation**		
Civil servant	10	7.6
Farming	57	43.5
Skilled labourer	8	6.1
Trading	32	24.4
Unskilled labourer	10	7.6
None	14	10.7

Most of the ulcers were on the legs of the patients (79.4%) and were present for 1–5 years (65.6%) with a mean duration of 5.0 ± 4.9 (Table 3).

**Table 3:** Characteristics associated with the ulcer among the patients

Variable	Frequency	Percentage
**Site of the ulcer**		
Arms	14	10.7
Back	6	4.6
Chest	2	1.5
Face	3	2.3
Genital	2	1.5
Leg	104	79.4
**Duration of ulcer**		
< 1 year	19	14.5
1–5 years	86	65.6
> 5 years	26	19.8
**Mean duration and standard deviation of ulcer**	5.0 ± 4.9	

Table 4 shows a comparison of the proportions of BU suspects that were identified before and after using the community volunteers. There was a significant increase in the proportion of BU suspects identified by the community volunteers in all 3 LGAs (Afikpo north = < 0.001, Abakaliki = 0.02, Ikwo = 0.001).

**Table 4:** Detection and Referral of Buruli ulcer for treatment before the study and after the study period

Local Government Area	No. of patients detected and referred Before	No. of patients detected and referred after	Test statistics Z test
Afikpo north	15	83	Z = 9.75 (58.39–80.49) *P* < 0.001
Abakaliki	10	20	Z = 2.32 (6.14, 60.52 *P* = 0.02
Ikwo	13	28	Z = 3.09 (14.9, 59.17) *P* = 0.001

The duration of the ulcer was associated with the detection and referral of the patients with higher levels of detection and referral among those whose ulcer had lasted 1–5 years in two of the LGAs (*P* < 0.000) (Table 5).

**Table 5:** Association between the independent variables and the detection and referral of patients with Buruli Ulcer

Variable	Abakaliki	Afikpo	Ikwo	Test statisticsχ^2^(*p* value)
**Age group**				
Children	2	8	5	2.563 (0.633)
Adults	17	67	22	
Older adults	1	8	1	
**Sex**				
Male	8	41	17	2.090(0.352)
Female	12	42	11	
**Duration of ulcer**				
< 1 year	2	11	6	23.645(*P* < 0.001)
1–5 years	8	65	13	
> 5 years	10	7	9	

## Discussion

In this study, we aimed to determine the effect of training community members as volunteers in the detection of BUs in community members and the referral of these patients to the hospital. We found a significant increase in the number of patients detected and referred after the study period across the three randomly selected communities.

Ulcers were more commonly located on the upper limbs among the respondents. The findings of study in Ghana were consistent with the findings of this study, however, with the inclusion of the lower extremities [10]. Conversely, this finding was at variance with several studies where ulcers were frequently found on the lower limbs [11–14]. Additionally, according to WHO, the lower limb is the most common site of occurrence of the ulcers [15]. Due to the hot climate conditions faced in several African countries, people tend to wear light clothing such as sleeveless shirts or shorts, especially during physical exertion activities like farming [10]. This could aid in long-term exposure to M. *ulcerans*, the infective agent of Buruli disease. Human infection through insect bites has been documented [16]. This reinforces the use of long and protective clothing in BU endemic areas.

Predominantly, more adults than children were detected as Buruli patients in this study. This is similar to the findings of a study done in Nigeria where people over 15 years of age were mostly affected [17]. This was also noted in an epidemiological report conducted in Australia [12]. In contrast, two studies in Ghana noted that children presented with more cases of BU than adults. Additionally, a cohort study in Benin has a conflicting result compared to this study’s finding [18]. It is noted that children are grossly affected in the BU endemic countries in Africa [19]. However, a study in Southwestern Ghana has documented that older people were likely to develop BUs, when the population was standardized for age [20]. This could be a plausible reason for the finding of age going in the opposite direction in this study. Most African countries have a younger population compared to developed countries with a high older population [21].

There was a significant association between the duration of ulcer and the place of residence. This could be explained by the role residence plays in the variability of geographical access to healthcare and the extent of endemicity of the disease in the locality [12]. In this study, the predominant cases with longer-lasting ulcers resided in communities known to be lowlands with bodies of water. This is likely to make health services inaccessible to them. Additionally, they are prone to frequent contact with unprotected water sources, which is an associated factor with BU. A study in Benin reported that access to unprotected water bodies, with emphasis on flooded lowlands, is implicated in a rise in BU cases [22]. Another implication of this finding could be the constant use of bodies of water for agriculture. Farming in swamps is a known predictor of BU disease [10]. Rice farming (which is usually done in swamps) was predominant among the respondents in this study.

There was a significant increase in the detection of cases with the use of community health volunteers in the early stages of the ulcer, compared to the pre-intervention period. This is in keeping with findings from research in Benin and Ghana, both in the West African region [7, 23]. It is known that the use of community volunteers facilitates early detection of cases and timely referrals and increases the knowledge and awareness of their preventive measures [24]. Across major infectious and non-infectious diseases, the role of community health volunteers cannot be overemphasized [24]. They are most impactful in low-income countries that are prone to limited staff strength in their health systems [25]. Most BU endemic countries belong to this category, and this affects the delivery of evidence-based interventions [26]. Based on trust, volunteers act as mediators between the formal health workers and the community members by breaking down the barriers to accessing health care, thereby improving their health-seeking pattern [27].

The major strength of our study lies in its novelty and the large sample size of respondents used in this study. Additionally, the findings from this study add to the growing evidence of the impact of community cealth volunteers on the control of BU. Notwithstanding, this study is limited by the absence of a control group. The use of self-reported data may have introduced recall bias. Furthermore, the limited number of sample sites may have affected the generalizability of our findings. This was mitigated by ensuring that responses were scrutinized before being captured. To increase the external validity of the findings, the study sites, though limited in number, were selected using probability techniques to ensure that they were representative of the study population.

## Conclusion

This study identifies the effect of community health volunteers in the detection and referral of BUs to the hospital. There was a significant increase in the number of cases detected and referred post-intervention. We recommend that program managers and stakeholders integrate and scale up the services of trained community health volunteers in the rapid detection of BU cases. We also advocate for the intensification of awareness and sensitization campaigns on BU preventive measures with a focus on those who reside in lowlands and water-logged areas that are predominantly farmers.

## Data Availability

The datasets used and/or analyzed during the current study are all available from the corresponding author on reasonable request.

## Abbreviations

IEC: Information, education and communication
LGA: Local government area
NTBLCP: National Tuberculosis leprosy and Buruli Ulcer control program
